# Fak56 functions downstream of integrin alphaPS3betanu and suppresses MAPK activation in neuromuscular junction growth

**DOI:** 10.1186/1749-8104-3-26

**Published:** 2008-10-16

**Authors:** Pei-I Tsai, Hsiu-Hua Kao, Caroline Grabbe, Yu-Tao Lee, Aurnab Ghose, Tzu-Ting Lai, Kuan-Po Peng, David Van Vactor, Ruth H Palmer, Ruey-Hwa Chen, Shih-Rung Yeh, Cheng-Ting Chien

**Affiliations:** 1 Institute of Molecular Biology, Academia Sinica, Taipei 115, Taiwan; 2 Institute of Molecular Medicine, National Taiwan University, Taipei 106, Taiwan; 3 Institute of Molecular Medicine, National Tsing Hua University, Hsinchu 300, Taiwan; 4 Umeå Center for Molecular Pathogenesis, Umeå University, Umeå, S-901 87, Sweden; 5 Department of Cell Biology and Program in Neuroscience, Harvard Medical School, Boston, Massachusetts 02115, USA; 6 Institute of Biological Chemistry, Academia Sinica, Taipei 115, Taiwan; 7 Indian Institute of Science Education and Research, 900, NCL Innovation Park, Homi Bhabha Road, Pune 411008, India

## Abstract

**Background:**

Focal adhesion kinase (FAK) functions in cell migration and signaling through activation of the mitogen-activated protein kinase (MAPK) signaling cascade. Neuronal function of FAK has been suggested to control axonal branching; however, the underlying mechanism in this process is not clear.

**Results:**

We have generated mutants for the *Drosophila FAK *gene, *Fak56*. Null *Fak56 *mutants display overgrowth of larval neuromuscular junctions (NMJs). Localization of phospho-FAK and rescue experiments suggest that Fak56 is required in presynapses to restrict NMJ growth. Genetic analyses imply that FAK mediates the signaling pathway of the integrin αPS3βν heterodimer and functions redundantly with Src. At NMJs, Fak56 downregulates ERK activity, as shown by diphospho-ERK accumulation in *Fak56 *mutants, and suppression of *Fak56 *mutant NMJ phenotypes by reducing ERK activity.

**Conclusion:**

We conclude that Fak56 is required to restrict NMJ growth during NMJ development. Fak56 mediates an extracellular signal through the integrin receptor. Unlike its conventional role in activating MAPK/ERK, Fak56 suppresses ERK activation in this process. These results suggest that Fak56 mediates a specific neuronal signaling pathway distinct from that in other cellular processes.

## Background

Formation and stabilization of neuronal synapses demands communication between pre- and post-synaptic partners, as well as signals from the extracellular matrix (ECM). These signals can reorganize local cytoskeletal structures or be transduced into the nucleus to regulate transcription, thereby modulating neuronal plasticity [1-3]. One major receptor family for ECM signals comprises the transmembrane protein integrins, which have been shown to play critical roles in sequential steps of neuronal wiring, such as in neurite outgrowth, axon guidance, and synaptic formation and maturation [4-7]. In *Drosophila*, various integrin subunits have been shown to function in motor axon pathfinding and target recognition, and synaptic morphogenesis at neuromuscular junctions (NMJs) [8-10]. Mutant analyses for the integrin subunits αPS3 and βPS indicate that integrin signaling is involved in synaptic growth and arborization of larval NMJs [8-10]. Although specific ECM signals for these integrin receptors are not clear, dynamic NMJ growth is regulated by heparan sulfate proteoglycans [11]. Also, the N-glycosaminoglycan-binding protein MTG (encoded by *mind the gap*), a pre-synaptic secreted ECM molecule, has been shown to shape the synaptic cleft and modulate post-synaptic differentiation [12].

Integrin signaling activities in cell adhesion, spreading and migration can be mediated by the non-receptor tyrosine kinase focal adhesion kinase (FAK) [13-15]. In these processes, FAK becomes activated when phosphorylated at tyrosine 397 (Y397) and associates with Src to form a dual kinase complex [14,16]. Activated Src phosphorylates FAK thereby creating a signaling cascade through Ras and mitogen-activated protein kinase (MAPK)/ERK [17-19]. Activated ERK can modulate focal contact dynamics during cell migration, as well as promote cell proliferation and survival. In *Drosophila *larval NMJ growth regulation, ERK is specifically activated by Ras and its activation downregulates the protein levels of the cell adhesion molecule Fasciclin II (FasII) at NMJs [20].

The significance of FAK in development has been revealed by *fak *knockout mice that are embryonic lethal at embryonic day 8.5 during gastrulation, consistent with its role in cell adhesion and migration [21]. FAK proteins are highly enriched in developing nervous systems, in particular in axonal tracks and growth cones [22-25]. Neuronal-specific depletion of *fak *leads to cortical abnormalities, revealing the requirement of FAK in neural development [26]. At the cellular level, ablation of *fak *in Purkinje cells induces axonal branching and synapse formation, and this FAK activity is suggested to be partially mediated through p190RhoGEF, which modulates cytoskeletal structure [27]. Inactivation of the only *Drosophila *FAK gene, *Fak56*, however, permits normal development and transduction of integrin signaling pathways [28]. A requirement for Fak56 in glial cells of the optic stalk has recently been reported, suggesting for the first time a role for FAK family kinase activity in *Drosophila *[29].

We have generated *Fak56 *mutants and identified a role for Fak56 in restricting NMJ growth. Analyses of genetic interactions suggest that Fak56 plays a conventional role in cooperation with Src to transduce integrin signaling. Fak56 is activated at NMJs, as shown by immunostaining for its phosphorylated form and this activation depends on the presence of the integrin βν subunit. ERK activation and FasII protein downregulation were observed at *Fak56 *mutant NMJs. The NMJ overgrowth phenotype and FasII downregualtion in *Fak56 *mutants can be suppressed by reducing ERK activity. The physiological output of the enlarged NMJ in *Fak56 *mutant displays increased synaptic response by nerve stimulation. These results suggest that Fak56 negatively regulates ERK activity and modulates synaptic plasticity at NMJs.

## Results

### Larval NMJ overgrowth in *Drosophila Fak56 *null mutants

**Figure 1:**
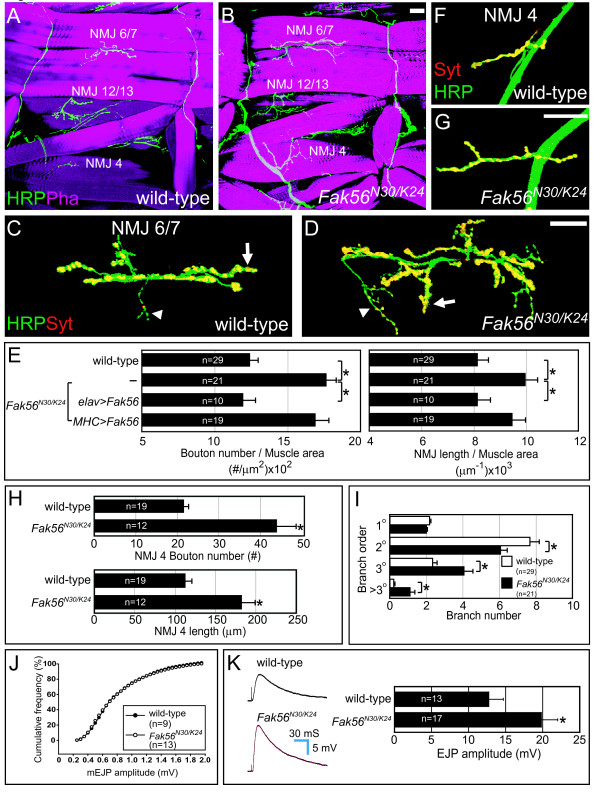
**NMJ overelaboration in *Fak56 *mutants.** (A, B) Muscular and axonal patterns in A3 segments shown by horseradish peroxidase (HRP)-labeled axonal branches of motor neurons (green), and Pha-labeled muscular pattern (magenta). NMJs 6/7, 12/13 and 4 are shown for wild-type (A) and *Fak56*^*N*30/*K*24 ^(B). In these and all other figures, scale bars represent 20 μm unless specifically indicated. (C, D) NMJ 6/7 phenotypes in A3 segments shown by HRP-labeled axons (green), Syt-labeled synaptic boutons (red) and Pha-labeled muscles (not shown). Arrows and arrowheads indicate type Ib and Is boutons, respectively. (E) Quantification of bouton numbers and total branch length that are normalized to total muscle 6/7 areas for wild-type, *Fak56*^*N*30/*K*24^, *Fak56*^*N*30/*K*24 ^*;elav*>*Fak56*, and *Fak56*^*N*30/*K*24^*;MHC*>*Fak56*. In this and all following quantifications, values are mean ± SEM, asterisks indicate *p *< 0.05 by Student's *t *test and sample numbers are within each bar. (F-H) NMJ 4 in A3 segments of wild-type (F) and *Fak56*^*N*30/*K*24 ^(G) are labeled as in (C, D), and quantifications of bouton number and total branch length are shown in (H). (I) Quantification for the number of branches at NMJ 6/7s of wild-type and *Fak56*^*N*30/*K*24^. Branches originating from the nerve entry point are primary (1°), and subsequent branches with at least three boutons are defined progressively with one higher order (2°, 3° or >3°). (J, K) Electrophysiological recording of postsynaptic currents in wild-type and *Fak56*^*N*30/*K*24 ^in 0.2 mM [Ca^2+^]. (J) Cumulative frequency plot to compare amplitudes of mEJPs in wild-type and *Fak56*^*N*30/*K*24^. (K) Representative traces (left panel) and mean peak amplitude (right panel) of EJPs in wild-type and *Fak56*^*N*30/*K*24^. Calibration: 30 ms, 5 mV for evoked release.

The Fak56 protein is highly expressed in the ventral nerve cord during embryonic stages [22,25]. To examine whether *Fak56 *has a role in NMJ formation, we dissected late third instar larvae from a transheterozygous *Fak56*^*N*30/*K*24 ^mutant that deletes the *Fak56 *gene and lacks *Fak56 *mRNA expression (Additional file 1A, B, and Additional file 1 legend for the generation of *Fak56 *mutants). This *Fak56 *null mutant was immunostained with horseradish peroxidase (HRP) in order to label axonal processes [30], and phalloidin (Pha) to label muscle fibers. No abnormality of motor axonal tracts could be detected, and the pattern and size of muscles were normal, in agreement with earlier observations [28]. However, a more detailed examination revealed that *Fak56 *null mutant NMJs were overelaborated in comparison to wild-type ones (Figure 1A, B). NMJs innervating muscles 6 and 7 (NMJ 6/7s) of abdominal segment 3 (A3) were analyzed by immunostaining for HRP and synaptotagmin (Syt) to label presynaptic boutons [31]. Altered branching patterns and ectopic synaptic boutons were observed, with increases in both Ib and Is boutons (arrows and arrowheads, respectively, in Figure 1C, D). Quantitatively, the number of synaptic boutons was increased by 44% and the total branch length increased by 22% when normalized to the total area of muscles 6 and 7 (quantified in Figure 1E). The *Fak56 *activity was not limited to NMJ 6/7s since NMJ 4s also displayed overgrowth phenotypes in both total branch length (62% increase) and bouton number (101% increase) (Figure 1F–H). Furthermore, previous reported *Fak56*^*CG*1 ^null mutants [28] also displayed a significant NMJ overgrowth phenotype when compared to wild-type control (Additional file 1C–E).

When scored for NMJ 6/7s, the altered branching pattern in *Fak56*^*N*30/*K*24 ^mutants showed secondary branch reduction by 21% but increases in higher-order branches (73% for tertiary branches and 424% for beyond tertiary; Figure 1I). The increase in higher-order branches was not caused by extension of multiple branches from single boutons, since a normal bifurcating pattern was observed.

To confirm that NMJ overgrowth phenotypes in the *Fak56*^*N*30/*K*24 ^mutant are due to the absence of *Fak56 *activity, a *UAS-Fak56 *transgene [25] was introduced. We found that neuronal expression of *Fak56 *with *elav-GAL4 *in the *Fak56*^*N*30/*K*24 ^mutant completely suppressed the NMJ phenotypes, as shown in assays for total branch length and bouton number of NMJ 6/7s. In contrast, *Fak56 *expression with the muscle-specific *MHC-GAL4 *failed to rescue *Fak56 *mutant phenotypes (Figure 1E). Taken together, these results suggest that *Fak56 *is specifically required in presynaptic neurons but not postsynaptic muscles to restrict NMJ growth. The exuberant NMJs in *Fak56 *null mutants were constructed normally, since molecular markers for various synaptic structures were expressed in a wild-type pattern (Additional file 2). Synaptic ultrastructure analyzed by transmission electron microscopy revealed no significant alternations in pre- and post-synaptic structures (Additional file 3).

### Synaptic transmission is affected in the *Fak56 *null mutant

To examine whether the enlarged NMJ in *Fak56 *null mutants is associated with functional changes in transmitter release, postsynaptic currents were recorded. In the null *Fak56*^*N*30/*K*24 ^mutant, no alteration was observed in the amplitude of spontaneous release of neurotransmitter or miniature junctional potentials (mEJPs) at a low Ca^2+ ^concentration (0.2 mM), as shown in the cumulative frequency plot (Figure 1J). Similar skews of distributions were measured for wild type and *Fak56*^*N*30/*K*24 ^(1.5 ± 0.1 in wild type and 1.7 ± 0.2 in *Fak56*^*N*30/*K*24^, 0.25 <*p *< 0.5 by Kruskal-Wallis *h *test). The variance/mean of mEJP amplitudes were also similar (0.28 ± 0.04 in wild type and 0.25 ± 0.04 in *Fak56*^*N*30/*K*24^, 0.25 <*p *< 0.5 by Kruskal-Wallis *H *test). The frequency of mEJP was not changed significantly (1.2 ± 0.2 Hz in wild type and 1.8 ± 0.3 Hz in *Fak56*^*N*30/*K*24^, *p *= 0.16, Student's *t*-test). Resting membrane potentials were similar in these measurements (-69.1 ± 1.9 mV in wild-type and -66.6 ± 1.5 mV in *Fak56*^*N*30/*K*24^, *p *= 0.32 by Student's *t*-test). However, the mean amplitude of nerve-evoked EJPs was significantly enhanced at *Fak56 *mutant NMJs compared to wild type (*p *= 0.026 by Student's *t*-test, Figure 1K; measurements were also performed at 1 mM [Ca^2+^]; Additional file 4). These data demonstrate a role of Fak56 in modulating the electrophysiological behavior of *Drosophila *NMJs.

### Involvement of integrin subunits αPS3 and βν in Fak56-regulated NMJ growth

**Figure 2:**
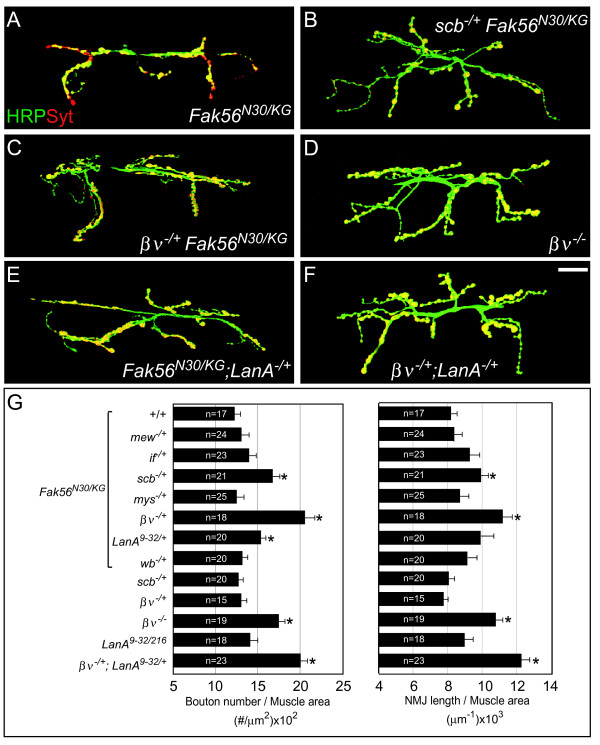
**Genetic interactions between *Fak56 *and integrin signaling pathway components during neuromuscular junction (NMJ) growth.** (A-F) Images of NMJ 6/7 are shown as described for Figure 1C, D. Hypomorphic *Fak56*^*N*30/*KG *^mutants showed a normal morphology (A), but one allele of *scb*^2 ^(B), *βν*^1 ^(C) or *LanA*^9–32 ^(E) in *Fak56*^*N*30/*KG *^induced dramatic NMJ growth. Overelaborated NMJs in transheterozygotes *βν*^1/2 ^(D) and *βν*^1/+^*;LanA*^9–32/+ ^(F) mutants are shown. **(G) **Quantification of NMJ 6/7 phenotypes for *Fak56*^*N*30/*KG*^, *mew*^1/+^*;Fak56*^*N*30/*KG*^, *if*^*k*27*e*/+^*;Fak56*^*N*30/*KG*^, *scb*^2/+ ^*Fak56*^*N*30/*KG*^, *mys*^1/+^*;Fak56*^*N*30/*KG*^, *βν*^1/+^*Fak56*^*N*30/*KG*^, *Fak56*^*N*30/*KG*^*;LanA*^9–32/+^, *wb*^4*Y*18/+ ^*Fak56*^*N*30/*KG*^, *scb*^2/+^, *βν*^1/+^, *βν*^1/2^, *LanA*^9–32/216 ^and *βν*^1/+^*;LanA*^9–32/+^. Asterisks indicate significant difference by Student's *t *test (*p *< 0.05) and error bars represent the standard error of the mean (SEM).

We then tested whether Fak56 mediates specific integrin activities at NMJs by genetic analysis. Integrin receptors are composed of heterodimeric α and β subunits [32]. In the *Drosophila *genome, there are five α subunits: αPS1 (encoded by *multiple edematous wings*, *mew*), αPS2 (*inflated*, *if*), αPS3 (*Vol *or *scb*), αPS4 and αPS5 (both αPS4 and αPS5 uncharacterized), and two β subunits (βPS (*myospheroid*, *mys*) and βν(*βν*)) [33-38]. We tested for possible genetic interactions between the available mutant alleles of integrin subunits and *Fak56*. In *Fak56*^*N*30/*KG *^hypomorphic animals, expression of *Fak56 *mRNA was reduced, but the NMJ appeared phenotypically normal (Additional file 1A, B; Figure 2A). However, when single mutant alleles of *scb*^2 ^and *βν*^1 ^were introduced into the *Fak56*^*N*30/*KG *^background, significant NMJ overgrowth was induced (Figure 2B, C). This overgrowth phenotype was not detected when *mew*^1^, *if*^*k*27*e *^and *mys*^1 ^were introduced (quantified in Figure 2G). As controls, larvae that were heterozygous for the *scb*^2 ^or *βν*^1 ^mutant alleles displayed normal NMJ bouton number and length (quantified in Figure 2G). These results suggest that compromised αPS3 or βν integrin signaling demands the full-strength of Fak56 activity to constrain NMJ growth. Since NMJ overgrowth has been observed for αPS3 but not βν mutants [9], we examined NMJ phenotypes in the viable *βν*^1/2 ^mutant. Strikingly, significant increases in both branch length and bouton number were detected, similar to those observed in *Fak56 *null mutant larvae (Figure 2D). In summary, these genetic analyses suggest that αPS3 and βν are the main integrin subunits in regulating Fak56 activity during NMJ growth.

The laminins are ECM components composed of heterotrimers of α,β and γ subunits, and are major signals for integrin receptors [39]. In *Drosophila*, *LanA *and *wing blister *(*wb*) encode two different α chains. We performed genetic interaction for both α chain mutants to test their involvement in *Fak56 *activity. Introducing one mutant allele of *LanA*^9–32 ^but not *wb*^4*Y*18 ^into the *Fak56*^*N*30/*KG *^hypomorphic background promoted a significant increase in the number of synaptic boutons (Figure 2E, G). The total NMJ length was also increased, although it was not significant (*p *= 0.37). While the hypomorphic *LanA*^9–32/216 ^mutant displayed normal NMJ phenotypes, transheterozygous *βν*^1/+^*;LanA*^9–32/+ ^displayed strong overgrowth phenotypes, with 61% increase in the bouton number and 32% increase in the total length compared to wild-type NMJs (Figure 2F, G). These results are consistent with a role for the α subunit LanA as a component of laminins to signal integrins during NMJ growth.

### Participation of Src in Fak56-regulated NMJ growth

**Figure 3:**
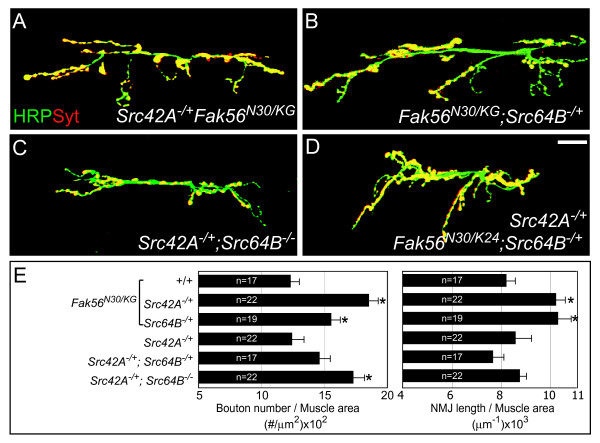
**Role of *Src *and its genetic interaction with *Fak56 *during neuromuscular junction (NMJ) growth.** (A-D) Images of NMJ 6/7 are shown as in Figure 1. *Fak56*^*N*30/*KG *^mutants carrying one allele of *Src42A*^*E*1 ^(A) or *Src64B*^*PI *^(B) displayed NMJ overgrowth phenotype. (C) NMJ phenotype in the severe *Src *mutant *Src42A*^*E*1/+^*;Src64B*^*PI*/*PI*^. (D) NMJ phenotype of the *Fak56*^*N*30/*K*24 ^null mutant was enhanced by removing both one *Src42A*^*E*1 ^and one *Src64B*^*PI *^allele. **(E) **Quantification of NMJ 6/7 phenotypes for *Fak56*^*N*30/*KG *^(the same set of data as in Figure 2G), *Src42A*^*E*1/+ ^*Fak56*^*N*30/*KG*^, *Fak56*^*N*30/*KG*^*;Src64B*^*PI*/+^, *Src42A*^*E*1/+^, *Src42A*^*E*1/+^*;Src64B*^*PI*/+ ^and *Src42A*^*E*1/+^*;Src64B*^*PI*/*PI*^. Note that *Src42A*^*E*1/+ ^and *Src42A*^*E*1/+^*;Src64B*^*PI*/+^show no significant alteration in NMJ phenotypes when compared to wild-type. Asterisks indicate significant difference by Student's *t *test (*p *< 0.05) and error bars represent the standard error of the mean (SEM).

Activated FAK forms a complex with Src, and the dual FAK-Src kinase complex induces downstream signaling [40]. To test whether Src is involved in Fak56-regulated NMJ growth, we performed genetic interactions between *Fak56 *and the *Drosophila Src *genes *Src42A *and *Src64B*. Reducing one gene dosage of either *Src42A *(*Src42A*^*E*1^) or *Src64B *(*Src64B*^*PI*^) in the *Fak56*^*KG*/*N*30 ^background displayed significant NMJ overgrowth, as scored for total branch length and bouton number (Figure 3A, B, E). Controls of *Src42A*^*E*1/+^*;Src64B*^+/+ ^and *Src42A*^*E*1/+^*; Src64B*^*PI*/+ ^in a wild-type background displayed no significant NMJ overgrowth (quantified in Figure 3E), suggesting that the efficiency of Src signaling at NMJs is dependant upon Fak56 activity in a dose-dependant manner. These results are consistent with a role for a FAK-Src complex in the restriction of NMJ growth.

We then tested whether severe *Src *mutants display NMJ growth defects. In the viable *Src42A*^*E*1/+^*; Src64B*^*PI*/*PI *^mutant that generates the least Src activity [41], the number of boutons was significantly increased and the total branch length was slightly enhanced (Figure 3C, E). To test whether Src has any contribution in the complete absence of Fak56 activity, we generated the combinatorial mutant *Src42A*^*E*1/+^*Fak56*^*N*30/*K*24^*;Src64B*^*PI*/+ ^and found that reducing the gene dosage of *Src *further increased the number of boutons in the *Fak56 *null mutant by 21%. In comparison to wild-type animal controls, *Src42A*^*E*1/+^*Fak56*^*N*30/*K*24^*;Src64B*^*PI*/+ ^mutants displayed an 80% increase in the bouton number and 25% increase in total branch length (Figure 3D). In summary, these genetic analyses suggest that Fak56 and Src have overlapping and distinct contributions in inhibiting NMJ growth.

### Activation of Fak56 at NMJs

**Figure 4:**
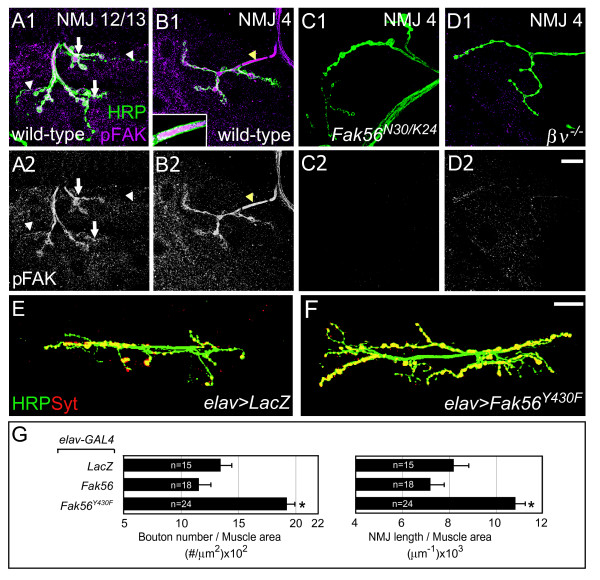
**Distribution and requirement of phospho-FAK (pFAK) at presynapses of neuromuscular junctions (NMJs).** (A-D) Active Fak56 (pFAK in magenta) recognized by anti-FAK [pY^397^] antibodies localized at presynapses of NMJ 12/13 (A) and NMJ 4 (B) in wild-type larvae, and was absent in *Fak56*^*N*30/*K*24 ^(C), and reduced in *βν*^1/1 ^(D), with co-stained horseradish peroxidase (HRP) in green. White arrows and arrowheads indicate Ib and Is boutons, respectively. Images in (A-D) come from a single section of the Z-stack confocal scanning. Note punctate distribution in muscles and strong expression in axonal trunks (yellow arrowhead in B1) surrounded by HRP membrane staining. The inset image in B1 is the inclusion of pFAK inside the incoming axon from a Z-stack section crossing the middle of the axon. (A2-D2) are diphospho-ERK (dpERK) images in white. (E-F) Images of NMJ 6/7 are shown as for Figure 1. Neuronal overexpression of LacZ control (E) or Fak56^*Y*430*F *^(F) by *elav-GAL4 *displays a NMJ overgrowth phenotype. (G) Quantification of NMJ 6/7 phenotypes for *elav*>*LacZ*, *elav*>*Fak56 *and *elav*>*Fak56*^Y430F^. Note that *elav*>*Fak56 *shows slight reduction in bouton number and NMJ length when compared to *elav*>*LacZ *control (E) but these reductions were not statistically significant (*p *= 0.413 for bouton number and *p *= 0.125 for branch length in Student's *t*-test). Asterisks indicate significant difference by Student's *t *test (*p *< 0.05) and error bars represent the standard error of the mean (SEM).

In mammals, activation of FAK and the FAK homolog Pyk2 proceeds with an auto-phosphorylation step at the conserved Y397 of FAK and Y402 of Pyk2 [16,40,42], which corresponds to Y430 in Fak56 [22,25,43]. To examine the activation of Fak56 at NMJs, we immunostained larval NMJs with the anti-FAK [pY^397^] antibody, which detects Fak56 activation at muscle attachment sites [28]. As shown for NMJ 12/13, phospho-FAK (pFAK) was expressed strongly in Ib boutons (white arrows in Figure 4A1) and weakly in Is boutons (white arrowheads). Expression at NMJ 4 was also prominent (Figure 4B1). In co-staining for HRP, the pFAK signals could be found within boutons and inter-bouton tracks (Figure 4A1, B1), suggesting a presynaptic activation of Fak56. Strong pFAK expression was also detected within the incoming axons that were co-labeled by HRP (yellow arrowhead and inset image in Figure 4B1). Cytosolic punctate staining was also present in muscles. In *Fak56*^*N*30/*K*24 ^null mutants, pFAK signals in axons, presynapses and muscles were completely absent (Figure 4C1, C2), confirming the specificity of the anti-pFAK antibody in detecting Fak56 activation signals.

In the *βν*^1/1 ^integrin mutant, the pFAK staining in presynapses was dramatically reduced while the muscle punctate staining pattern was still retained (Figure 4D1, D2), indicating that integrin signaling mediated by the βν subunit is required for Fak56 activation in presynapses of NMJs. Taken together with the requirement of βν in restricting NMJ growth, these results suggest the presynaptic activation of Fak56 in restricting NMJ growth. To test this, the autoactivation site Y430 in Fak56 was mutated to phenylalanine to generate the *UAS-Fak56*^*Y*430*F *^transgene. When ectopically expressed in neurons by *elav-GAL4*, *Fak56*^*Y*430*F *^induced significant NMJ overgrowth phenotypes (Figure 4F). As a control, the wild-type *Fak56 *transgene caused slight but no significant reduction in NMJ growth (quantified in Figure 4G). This dominant negative effect by the *Fak56*^*Y*430*F *^mutant suggests that phosphorylation at Y430 in the presynapse is critical for normal Fak56 function to constrain NMJ growth.

### Fak56 suppresses MAPK/ERK activation at NMJs

**Figure 5:**
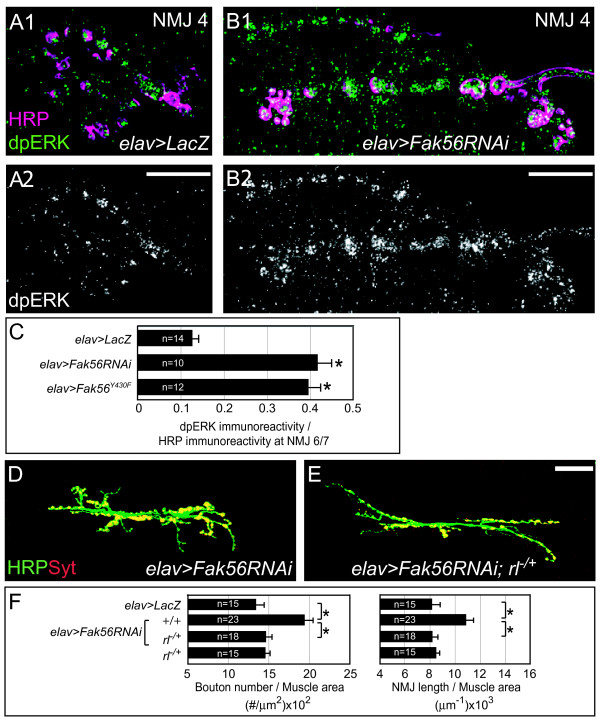
**Suppression of ERK activity by Fak56 during neuromuscular junction (NMJ) growth.** (A, B) Immunostaining of *elav*>*LacZ *(A1) and *elav*>*Fak56RNAi *(B1) for diphospho-ERK (dpERK; green) and horseradish peroxidase (HRP; magenta). A single section of image is shown. Punctate expression of dpERK was observed in presynaptic boutons and enhanced in the enlarged NMJ 4 in *elav*>*Fak56RNAi*. (A2, B2) Only dpERK expression is shown. Scale bars are 10 μm. (C) Quantification of relative immunoreactivities of dpERK to HRP within the presynaptic zone. Note that *elav*>*Fak56RNAi *and *elav*>*Fak56*^*Y*430*F *^had 3.3- and 3.1-fold increases compared to *elav*>*LacZ*. (D, E) Images of NMJ 6/7 are shown as described in Figure 1. (E) Depletion of Fak56 activity in *elav*>*Fak56RNAi *results in NMJ overgrowth, which can be suppressed by the *rl*^*EMS*698 ^allele (F). (F) Quantification of NMJ 6/7 phenotypes for the control *elav*>*LacZ*, *elav*>*Fak56RNAi*, *elav*>*Fak56RNAi;rl*^*EMS*698/+ ^and *rl*^*EMS*698/+^. Note that the *rl*^*EMS*698 ^allele suppressed NMJ phenotypes in *elav*>*Fak56RNAi*. Asterisks indicate significant difference by Student's *t *test (*p *< 0.05) and error bars represent the standard error of the mean (SEM).

To further investigate the role of Fak56 at the presynapse, we generated an *RNAi *transgene to deplete *Fak56 *expression (see Materials and methods and Additional file 1F). Expression of the *Fak56RNAi *transgene in presynapses (*elav*>*Fak56RNAi*) resulted in an increase in both total branch length and bouton number of NMJs compared to the *elav*>*LacZ *control (Figure 5E, G). In contrast, *Fak56 *depletion in muscles using *MHC-GAL4 *retained normal NMJ phenotypes (not shown).

It has been shown that presynaptic ERK activation promotes larval NMJ growth [20]. We then tested whether Fak56 had an effect on ERK activation at NMJs, which can be monitored by immunostaining for diphospho-ERK (dpERK) [44]. The expression of dpERK was detected in punctate patterns in some but not all boutons (Figure 5A1, A2) [20].

We then examined whether dpERK expression at NMJs was altered by presynaptic depletion of Fak56 using RNA interference (RNAi). In *elav*>*Fak56RNAi*, dpERK expression was highly enriched in almost all boutons at the enlarged NMJ (Figure 5B, B1). To quantify the difference among wild-type and *Fak56 *mutants, the level of dpERK immuno-reactivity within the presynaptic region was normalized to that of co-stained HRP. We found that in *elav*>*Fak56RNAi *the ratio was increased by 3.3-fold when compared to that in *elav*>*lacZ*. Consistently, neuronal expression of the dominant-negative Fak56^Y430F ^also resulted in strongly enhanced dpERK expression to 3.1-fold (Figure 5C). The enhancement in dpERK expression levels in both approaches to block Fak56 function suggests that Fak56 activation suppresses ERK signaling in presynaptic boutons.

To test whether NMJ overgrowth phenotypes in *Fak56 *mutants were caused by the increased ERK activity, one wild-type allele of the ERK gene *rolled *(*rl*) [45] was replaced with the null allele *rl*^*EMS*698 ^[46] in *elav*>*Fak56RNAi *larvae. The control heterozygous *rl*^*EMS*698/+ ^larvae displayed normal NMJ phenotypes. However, reduction of ERK gene dosage by 50% completely suppressed the NMJ overgrowth phenotypes observed in *elav*>*Fak56RNAi *(Figure 5D–F). The BMP/Gbb signaling pathway also promotes NMJ growth [47]. We then tested whether the BMP/Gbb pathway would have a similar regulation in *Fak56 *mutant NMJs. Three mutants in the BMP/Gbb signaling pathway components were tested for potential genetic interactions with Fak56 but failed to significantly modify NMJ phenotypes in *elav*>*Fak56RNAi *larvae (Additional file 5). Taken together, these results suggest that Fak56 specifically downregulates the growth-promoting ERK signaling during NMJ growth.

### Fak56 modulates IgCAM FasII levels at NMJs

**Figure 6:**
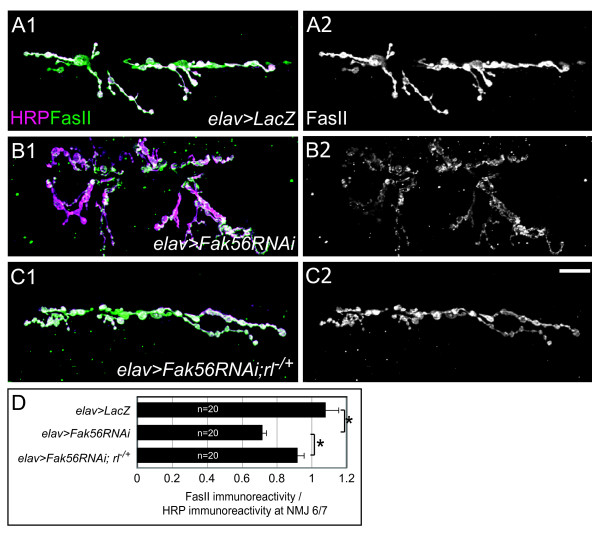
**Modulation of Fasciclin II (FasII) levels by Fak56.** (A-C) Expression of FasII (green) at neuromuscular junction (NMJ) 6/7 in *elav*>*LacZ *(A1), *elav*>*Fak56RNAi *(B1), and *elav*>*Fak56RNAi;rl*^*EMS*698/+ ^(C1). Co-stained horseradish peroxidase (HRP) is in magenta. (A2-C2) Only FasII staining is shown. Images in (A-C) come from a single section of the Z-stack confocal image. (D) Quantification of FasII levels relative to HRP immunoreactivity shown in (A1-C1). Note that *elav*>*Fak56RNAi *had a 33.5% reduction compared to *elav*>*LacZ*, which was restored significantly by removing one copy of *rl *in *elav*>*Fak56RNAi;rl*^*EMS*698/+^. Asterisks indicate significant difference by Student's *t *test (*p *< 0.05) and error bars represent the standard error of the mean (SEM).

It has been shown that ERK signaling regulates NMJ growth through the modulation of the protein levels of the cell-adhesion protein FasII [20]. At NMJs, FasII protein levels are inversely correlated with ERK activation. In *elav*>*Fak56RNAi *mutants, the NMJ FasII level was reduced (Figure 6B1). Using the *elav*>*LacZ *as the reference, a 33.5% reduction in the ratio of the FasII level to the HRP level was detected (Figure 6A1, D). Comparison of FasII expression between wild type and *Fak56*^*N*30/*K*24 ^also revealed a 26.6% reduction in the *Fak56 *mutant (images not shown). Analyses of these two mutants suggest that Fak56 activity in presynapses is required for the full expression of FasII at NMJs. To examine whether Fak56-regulated FasII expression is mediated through ERK, the FasII protein level was examined in *elav*>*Fak56RNAi;rl*^*EMS*698/+^. We found that the FasII protein level at NMJs of *elav*>*Fak56RNAi *was significantly restored by introducing the *rl*^*EMS*698 ^allele, with only 14.9% reduction compared to *elav*>*LacZ *(Figure 6C1, D). These results suggest that Fak56 regulation of FasII expression at NMJs is at least partially mediated by ERK.

## Discussion

**Figure 7:**
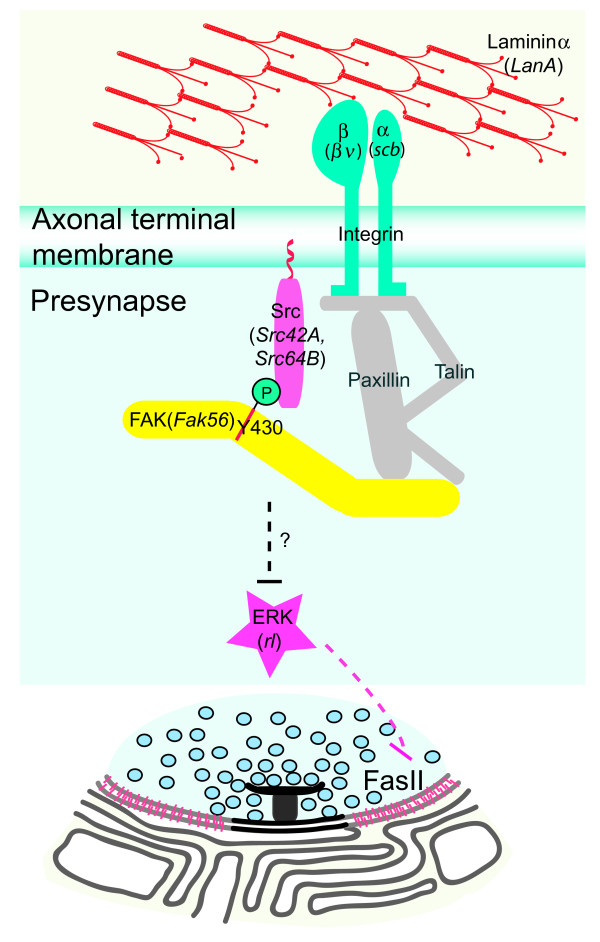
**Model to depict Fak56 and ERK signaling and Fasciclin II (FasII) protein levels at neuromuscular junctions (NMJs).** In restricting NMJ growth, the extracellular matrix signal laminin including the α subunit LanA is received by integrin receptors, including αPS3 and βν subunits. This signal is transduced through the association between Fak56 and Src, and in this process phosphorylation of Y430 Fak56 is essential. Activated Fak56 mediates signaling through suppressing ERK activation at NMJs and consequently upregulates the FasII protein level at NMJs, leading to the inhibition of NMJ growth. Those molecules (shown in grey) were not tested in this study.

Growth of the stereotypical NMJs during larval stages is tightly regulated by signaling pathways that either promote or inhibit terminal branching, bouton addition and active zone formation [20,47-50]. In this study, we have identified an inhibitory role of the non-receptor tyrosine kinase FAK in the regulation of NMJ growth. The *Drosophila *FAK is required in presynaptic boutons for the growth process, where it functions in concert with the non-receptor tyrosine kinase Src. As evidenced by our genetic analysis, Fak56 plays a conventional role in mediating signal from the integrin receptors that mainly consist of αPS3 and βν subunits. Noncanonically, Fak56 suppresses MAPK/ERK activity in restricting synaptic elaboration. In support of this context-specificity of FAK activity, we have noted no gross changes in the dynamic patterns of ERK activation during *Fak56 *mutant embryogenesis (Additional file 6). Our data suggest that Fak56 activity inhibits ERK signaling in restricting synapse growth (Figure 7).

The importance of FAK in regulating axonal branching of motor neurons in *Drosophila *is revealed in this study and has been shown in Purkinje cells [27]. FAK activity in Purkinje cells has been attributed partially to the recruitment of p190RhoGEF during axonal branching and growth. In integrin-mediated cell adhesion, Rho activity is initially downregulated and followed by sustained activation, leading to actin reorganization [51]. In response to integrin signaling, the initial downregulation of Rho activity requires the activation of p190RhoGAP by tyrosine phosphorylation and association with SH2 domain-containing p120RasGAP, thus providing an alternative link between FAK and the Ras-MAPK pathway. Future studies on the characterization of the p190RhoGAP-p120RasGAP complex in NMJ development should illuminate how FAK regulates synaptic growth and plasticity.

ERK signaling regulates the protein levels of the cell adhesion molecule FasII at NMJs [20]. Homophilic interaction of FasII-like IgCAMs regulates axon pathfinding, target recognition, and synapse formation and remodeling [52-57]. At *Drosophila *NMJs, FasII is involved in synaptic formation and maintenance [52,53,56,57]. Different levels of FasII play different roles in NMJ formation. While the basal level is essential to form the synaptic structure, a higher-level of FasII protein restricts NMJ growth. We found that Fak56 regulates the high level of FasII at NMJs and this regulation could be accounted for by a suppression of ERK activity. Therefore, in NMJ growth regulation, the cell-matrix interaction mediated by integrin signaling cross-talks with FasII-dependent cell-cell adhesion between pre- and post-synaptic partners (Figure 7).

Previous analysis of the activity of the *Drosophila *integrin αPS3 in the viable *Vol *allele suggested that αPS3 regulates NMJ elaboration, synaptic transmission and plasticity [9]. Lack of αPS3 induces moderate NMJ overgrowth with increases in higher-order branches and boutons, similar to what were observed in *Fak56 *mutants. In our analysis, *βν *genetically interacts with the *Fak56 *mutant and the *βν *mutant NMJ displays an overgrowth phenotype as well, suggesting that βν may be the major β subunit forming integrin heterodimers with αPS3 to restrict NMJ growth. The integrin subunits αPS1, αPS2 and βPS are also expressed at NMJs, and alteration of βPS activity affects NMJ morphology [10]; it is thus foreseeable that multiple modes of integrin signaling pathways regulate NMJ growth.

Laminins are the major component of the ECM and are involved in NMJ synaptic formation and maintenance [58]. Functional laminins are heterotrimers composed of α, β and γ chains, and different chain combinations contribute to laminin diversity. Laminins 4, 9 and 11 are composed of the same β2 and γ1 chain but differ in the α chain (α2, α4 and α5, respectively) and have been shown to localize in synaptic clefts of the mammalian neuromuscular system [59]. In an *in vitro *culture system, laminin 11 with the α5 subunit serves as a stop signal in motor axon outgrowth [59]. In *Drosophila*, LanA is most homologous to mammalian α3 and α5 subunits. *LanA *genetically interacts with *Fak56 *and *βν *mutants and may serve as the conserved component of the stop signal to restrict NMJ elaboration.

## Conclusion

FAK activation by integrins regulates various cellular processes, and in many cases can be accounted for by an activation of Ras through the recruitment of the GRB2-SOS complex [14]. In our study, Fak56 activity restricts NMJ synaptic elaboration by inhibiting the ERK signaling cascade. This noncanonical link between FAK activity and ERK signaling might be cell-context specific, such as in neurons, or even subcellular site-specific, such as at synapses. Vol (αPS3) functions in the process of learning and memory [35], and can act as the FAK upstream regulator with the same regulatory link proposed here (Figure 7). FAK has been suggested as a putative therapeutic target for its role in tumor cell invasion and metastasis [13,15,60-62]. The neuronal-specific nonconventional link between FAK and ERK proposed in this study may have implications in cancer biology and therapy.

## Materials and methods

### Fly stocks

Flies were reared at 25°C except where specifically indicated. Wild-type flies used in this study were the *w*^1118 ^strain. Mutant alleles *Fak56*^*KG*00304^, *mew*^1^, *if*^*k*27*e*^, *scb*^2^, *mys*^1^, *Src42A*^*E*1^, *Src64B*^*PI *^and *rl*^*EMS*698 ^were obtained from the Bloomington stock center. *βν*^1^, *βν*^2 ^[34], *LanA*^9–32^, *LanA*^216 ^[63] and *wb*^4*Y*18 ^[64] have been previously described. The various *Fak56 *alleles used in this study are described in detail in Additional file 1. The transgenic lines *elav-GAL4 *(X) (used in neuronal *Fak56 *knockdown and overexpression), *elav-GAL4 *(III) (used in neuronal *Fak56 *rescue), and *UAS-LacZ *were obtained from the Bloomington stock center.*UAS-Fak56 *[28] and *MHC-GAL4 *[65] have been described previously. The *pUAST-Fak56RNAi *construct was generated by subcloning two inverted *Fak56 *cDNA fragments (base pairs 629–1177) into the *pUAST *vector and the knockdown effect was examined (Additional file 1F).*pUAST-Fak56*^*Y*430*F *^flies were generated from *pUAST-Fak56 *by PCR based site-directed mutagenesis. To enhance the *Fak56RNAi *transgene expression, embryos from the *elav*-*GAL4 *(X) and *pUAST-Fak56RNAi *cross were collected for 6 hours, kept at 25°C for 45 hours and shifted to 30°C until late third instar.

### Immunostaining

In all experiments, wandering late third instar larvae were dissected for analysis of NMJ phenotypes. After dissection, tissues were incubated in fixative solution (4% formaldehyde in 1× phosphate-buffered saline) for 20 minutes. For immunostaining, primary antibodies used were against synaptotagmin (mouse, 1:25; DHSB, Iowa City, IA, USA), HRP conjugated with TRITC (rabbit, 1:100; Jackson ImmunoResearch, West Grove, PA, USA), FAK [pY^397^] (rabbit, 1:50; Biosource-Invitrogen, Carlsbad, CA, USA), FasII (1D4, 1:100; DHSB) and dp-ERK-1/2 (mouse, 1:20; Sigma-Aldrich, St. Louis, MO, USA). Alexa 488-, Cy3- and Cy5-conjugated secondary antibodies and TRITC-phalloidin were used (Jackson ImmunoResearch).

### Image processing and presentation

Confocal images were acquired using a Zeiss LSM 510 Meta and processed using Adobe Photoshop CS. Images for quantification of NMJ branch length and bouton number were from a projection of 10 z-sections of 6.5–8 μm in total. To quantify the NMJ length and muscle area, the images were analyzed by a measurement tool in Zeiss LSM Image Examiner. For quantification of signal intensity at NMJs, images were acquired under the same scanning parameters. NMJs were outlined and the signal intensity was calculated by histogram analysis in Adobe Photoshop CS.

### Electrophysiological recording

For sample preparation, dissected larval body walls (including the central nervous system and motor axons) were exposed in cold (4°C) HL3.1 Ca^2+ ^free saline (70 mM NaCl, 5 mM KCl, 4 mM MgCl_2_, 10 mM NaHCO_3_, 5 mM trehalose, 115 mM sucrose, 5 mM HEPES pH 7.2) [66]. Experiments were performed on muscle 6 of segment A3 in late third instar larvae. The segmental nerve was cut near the ventral ganglion. Preparations were then incubated in HL3.1 saline containing 0.2 or 1 mM CaCl_2 _for electrophysiological experiments at room temperature (22°C). For stimulation and recording, a glass microelectrode (30–50 MO in resistance) filled with 3 M KCl was impaled in the sixth muscle of the third abdominal segment to record the EJPs. The mEJPs occurring in the background within 200 seconds were obtained without any stimulation on the segmental nerve. To evoke an EJP, the segmental nerve was stimulated every 30 seconds through the cut end with a suction electrode with 0.1 ms of pulse duration at 2 times the threshold voltage. Once the threshold voltage was reached, the size of EJPs remained unchanged despite the increase in stimulating voltage. Signals were digitized at 64 KHz by a PCI-6221 data-acquisition card (National Instrument, Austin, Texas, USA), and saved on an IBM compatible PC for analysis.

## Abbreviations

dpERK: diphospho-ERK; ECM: extracellular matrix; FAK: focal adhesion kinase; FasII: Fasciclin II; HRP: horseradish peroxidase; MAPK: mitogen-activated protein kinase; mEJP: miniature junctional potential; NMJ: neuromuscular junction; pFAK: phospho-FAK; RNAi: RNA interference.

## Competing interests

The authors declare that they have no competing interests.

## Authors' contributions

PIT designed the study, wrote the manuscript, and performed and participated in all experiments. HHK, YTL and SRY participated in the electrophysiological experiments and analysis. CG and RHP designed, manufactured, and supplied the *Fak56 point *mutation and *Fak56RNAi *constructs, performed dpERK embryonic staining, and contributed to manuscript revisions. AG and DVV helped to characterize the NMJ phenotype of *Fak56*^*CG*1^. TTL and KPP helped to analyze the synaptic markers at NMJ of *Fak56 *mutants. RHC helped revise the manuscript and provided suggestions with regard to signal transduction and intellectual input for the study. CTC participated in the overall design and coordination of the study and helped to write the manuscript. All authors have read and approved the final manuscript.

## Supplementary Material

Additional file 1 *Fak56 *mutant alleles and expression. This file describes (A) the *Fak56 *locus and the generation of *N30 *and *K24 *deletions from *KG00304 *P-element insertion, (B) the expression of *Fak56 *mRNA in different *Fak56 *allele combinations, (C, D) HRP staining for wild-type and *Fak56*^*CG*1 ^NMJs, (E) quantifications for NMJ phenotypes, and (F) the knock-down effect of *Fak56RNAi*. Click here for file

Additional file 2 Expressions of NMJ proteins in *Fak56*^*null*^. This file describes identical expressions of Dlg (A, B), Brp (C, D), dPak (E, F), GluIIA (G, H) and Fustch (I, J) at wild-type and *Fak56*^*N*30/*K*24 ^NMJs. Click here for file

Additional file 3 Ultrastructures of *Fak56*^*N*30/*K*24 ^synapses. Electron micrographs of cross-sections through a type-I bouton of muscle 6/7 in wild-type (A) and *Fak56*^*N*30/*K*24 ^(B) larvae. Quantitative analyses reveal no difference for synaptic unltrastructures (C). Click here for file

Additional file 4 Electrophysiological recording of postsynaptic currents from wild-type and *Fak56*^*N*30/*K*24 ^in 1 mM [Ca^2+^]. (A) Cumulative frequency plot reveals a significant shift in the distribution of mEJP amplitudes. (B) Representative traces and mean amplitudes of EJPs in wild-type and *Fak56*^*N*30/*K*24^. Click here for file

Additional file 5 BMP/Gbb signaling-independent mechanism of Fak56 in NMJ growth. No alternations of NMJ phenotypes were detected by introducing mutant alleles (*sax*^4^, *wit*^*A*12 ^and *med*^13^) for BMP signaling components into *elav>Fak56RNAi*. Click here for file

Additional file 6 ERK phosphorylation in *Fak56*^*CG*1 ^mutant embryos. Expressions of phospho-ERK appear grossly normal during *Drosophila *embryogenesis. Click here for file
