# Supplementary material for: Fak56 functions downstream of integrin alphaPS3betanu and suppresses MAPK activation in neuromuscular junction growth
Source: Neural Dev. 2008 Oct 16;3:26. doi: 10.1186/1749-8104-3-26 (PMC2576229; doi:10.1186/1749-8104-3-26)
Supplement: Additional file 1 — Fak56 mutant alleles and expression. This file describes (A) the Fak56 locus and the generation of N30 and K24 deletions from KG00304 P-element insertion, (B) the expression of Fak56 mRNA in different Fak56 allele combinations, (C, D) HRP staining for wild-type and Fak56CG1 NMJs, (E) quantifications for NMJ phenotypes, and (F) the knock-down effect of Fak56RNAi. [file 1749-8104-3-26-S1.pdf]

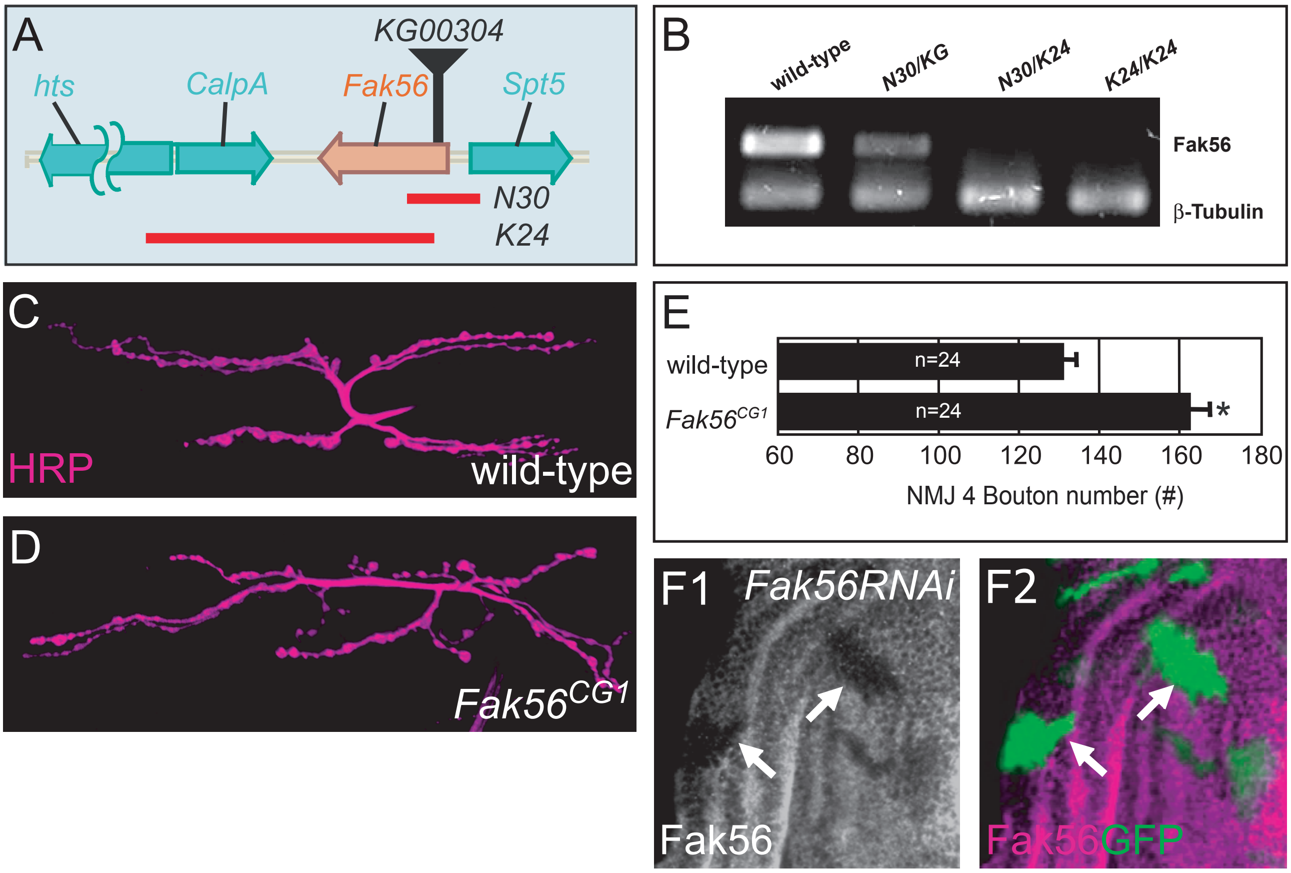

Additional file 1. *Fak56* mutant alleles and expression

(A) Illustration of the *Fak56* locus and flanking genes (indicated by arrowed boxes). The *KG00304* insertion was mapped at the 5'-UTR region of *Fak56* [28] and was used to generate imprecise excision lines. From 2,100 independent lines, 34 lines that deleted *Fak56* and the neighboring *Spt5* gene were obtained. The lethal *N30* allele, which deletes a 3030-bp chromosomal region including 2157 bp of *Fak56*, 240 bp of the shared promoter region, and 633 bp of *Spt5*. *N30* homozygotes were embryonic lethal due to the lack of *Spt5* activity since *N30* failed to complement *Spt5* null alleles (P.I. Tsai and C.T. Chien, unpublished results). Male-specific recombination [1] was used to generate the *K24* allele. Male flies carrying $Fak56^{KG}$*/Sp Pin; TM3 Sb Δ2-3/+* were crossed with *Pin/CyO* females, and progenies of *Sp* $Fak56^{KG}$*/CyO* were further analyzed. Of 20 lines analyzed, 7 alleles retained the P-element insertion at the 5'-UTR of *Fak56*, suggesting a recombination event. Of them, the *K24* allele included a 13 kb deletion, from the 3' end of P-element to +1191 bp downstream of the *hts* transcriptional start site, which deletes *Fak56*, C*alpA* and *hts*. *K24* homozygotes were adult-viable. The viability is consistent with the report for a specific *Fak56* mutant [28], and the female-sterility could be due to the absence of *hts* activity [2].

(B) *Fak56* mRNA expression in late 3rd instar larvae examined by RT-PCR. *Fak56* mRNA expression was reduced in the $Fak56^{N30/KG}$ hypomorphic mutant and absent in $Fak56^{N30/K24}$ and $FAK56^{K24/K24}$ null mutants. Expression of β*Tub* mRNA served as internal controls.

(C-D) NMJ 6/7 phenotypes shown by HRP-labeled axonal branches (magenta). Hypergrowth of NMJs is shown in $Fak56^{CG1}$ (D), as compared to wild-type (C).

(E) Quantification of NMJ 6/7 bouton numbers in wild-type and $Fak56^{CG1}$. Note that a 22% significant bouton number incense observed in $Fak56^{CG1}$ as compared to wild-type.

(F) Clonal expression of *Fak56RNAi* induced by *hs-Flp; Actin>CD2>GAL4 UAS-GFP* [3]. The clones expressing Fak56 RNAi are marked with GFP. In clones expressing *Fak56RNAi* (green in F2), a reduction of Fak56 expression (white in F1 and magenta in F2) can be detected (arrows).

References:

1. McCarron M, Duttaroy A, Doughty G, Chovnick A: **Drosophila P element transposase induces male recombination additively and without a requirement for P element excision or insertion.** *Genetics* 1994, **136:**1013-1023.
2. Yue L, Spradling AC: **hu-li tai shao, a gene required for ring canal formation during Drosophila oogenesis, encodes a homolog of adducin.** *Genes Dev* 1992, **6:**2443-2454.
3. Pignoni F, Zipursky SL: **Induction of Drosophila eye development by decapentaplegic.** *Development* 1997, **124:**271-278.
