# Supplementary material for: Fak56 functions downstream of integrin alphaPS3betanu and suppresses MAPK activation in neuromuscular junction growth
Source: Neural Dev. 2008 Oct 16;3:26. doi: 10.1186/1749-8104-3-26 (PMC2576229; doi:10.1186/1749-8104-3-26)
Supplement: Additional file 2 — Expressions of NMJ proteins in Fak56null. This file describes identical expressions of Dlg (A, B), Brp (C, D), dPak (E, F), GluIIA (G, H) and Fustch (I, J) at wild-type and Fak56N30/K24 NMJs. [file 1749-8104-3-26-S2.pdf]

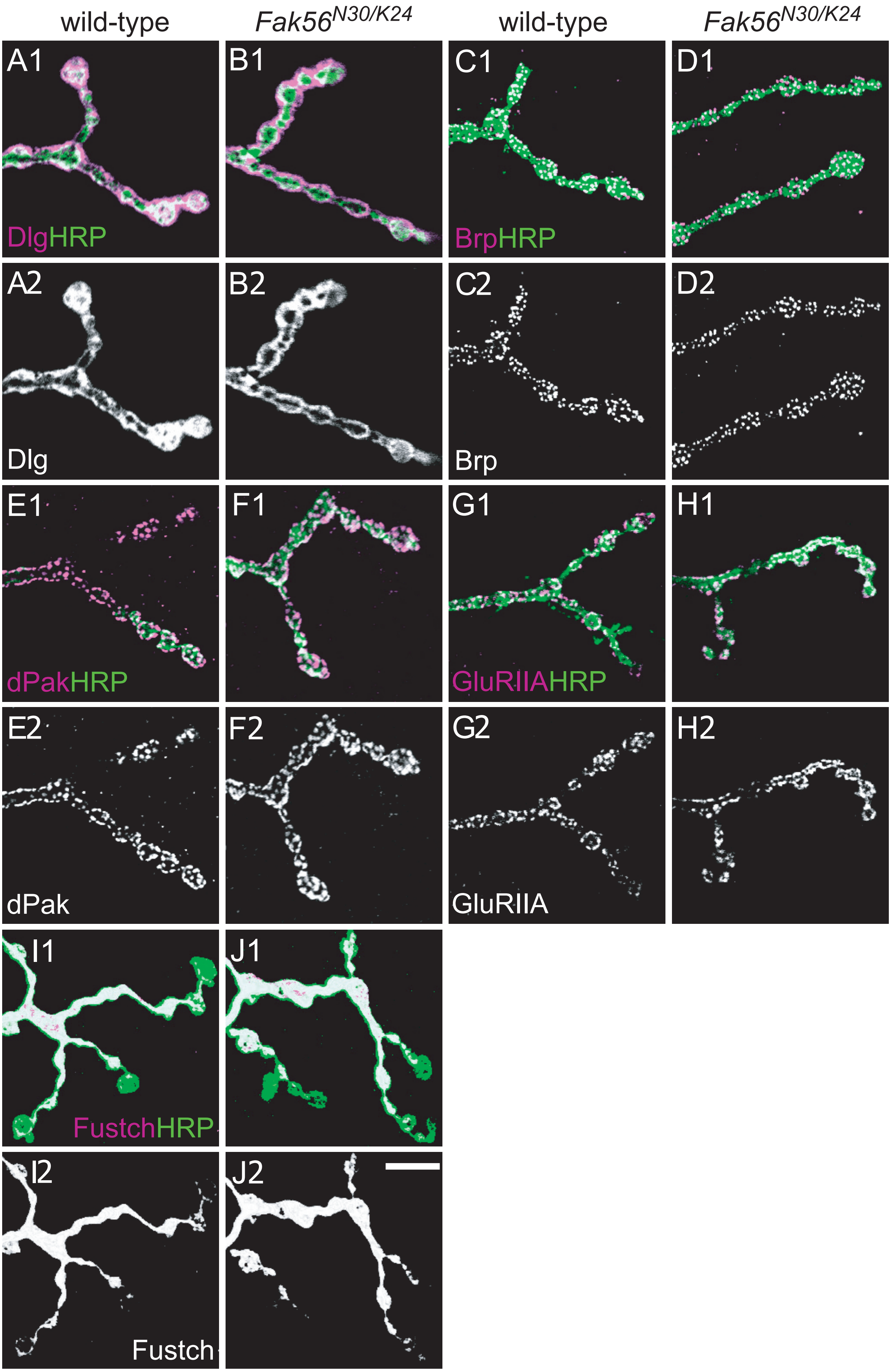
wild-type
Fak56^N30/K24
wild-type
Fak56^N30/K24
A1
DlgHRP
B1
C1
BrpHRP
D1
A2
Dlg
B2
C2
Brp
D2
E1
dPakHRP
F1
G1
GluRIIAHRP
H1
E2
dPak
F2
G2
GluRIIA
H2
I1
FustchHRP
J1
I2
Fustch
J2

Additional file 2. Expression of NMJ proteins in *Fak56*^{null}
(A-H) Expressions of Dlg (magenta in A1 and B1, white in A2 and B2) in postsynapses, Brp (magenta in C1 and D1, white in C2 and D2) at presynaptic active zones, dPak (magenta in E1 and F1, white in E2 and F2) and GluIIA (magenta in G1 and H1, white in G2 and H2) in postsynaptic active zones, and Fustch (magenta in I1 and J1, white in I2 and J2) for synaptic microtubules are shown for wild-type (A1, C1, E1, G1 and I1) and *Fak56*$^{N30/K24}$ (B1, D1, F1, H1 and J1) at NMJ 4s that were co-stained with HRP (green). The expression patterns and intensities of these proteins were identical between wild-type and *Fak56*$^{N30/K24}$. Images in (A-H) come from single section of the Z-stack confocal image and in (I and J) come from Z-stack projection. For GluRIIA staining, Bouin's fixative solution (Sigma Chemical Co.) was used [1]. Primary antibodies were used against Dlg (4F3, 1:100, DHSB), Brp (nc82, 1:100, DHSB), GluRIIA (8B4D2, 1:100, DHSB), dPAK (rabbit, 1:4000) and Fustch (22C10, 1:100, DHSB). Normal NMJ bouton ultrastructure in *Fak56*$^{N30/K24}$

References:

1. Marrus SB, Portman SL, Allen MJ, Moffat KG, DiAntonio A: **Differential localization of glutamate receptor subunits at the Drosophila neuromuscular junction.** *J Neurosci* 2004, **24:**1406-1415.
