# Supplementary material for: Fak56 functions downstream of integrin alphaPS3betanu and suppresses MAPK activation in neuromuscular junction growth
Source: Neural Dev. 2008 Oct 16;3:26. doi: 10.1186/1749-8104-3-26 (PMC2576229; doi:10.1186/1749-8104-3-26)
Supplement: Additional file 3 — Ultrastructures of Fak56N30/K24 synapses. Electron micrographs of cross-sections through a type-I bouton of muscle 6/7 in wild-type (A) and Fak56N30/K24 (B) larvae. Quantitative analyses reveal no difference for synaptic unltrastructures (C). [file 1749-8104-3-26-S3.pdf]

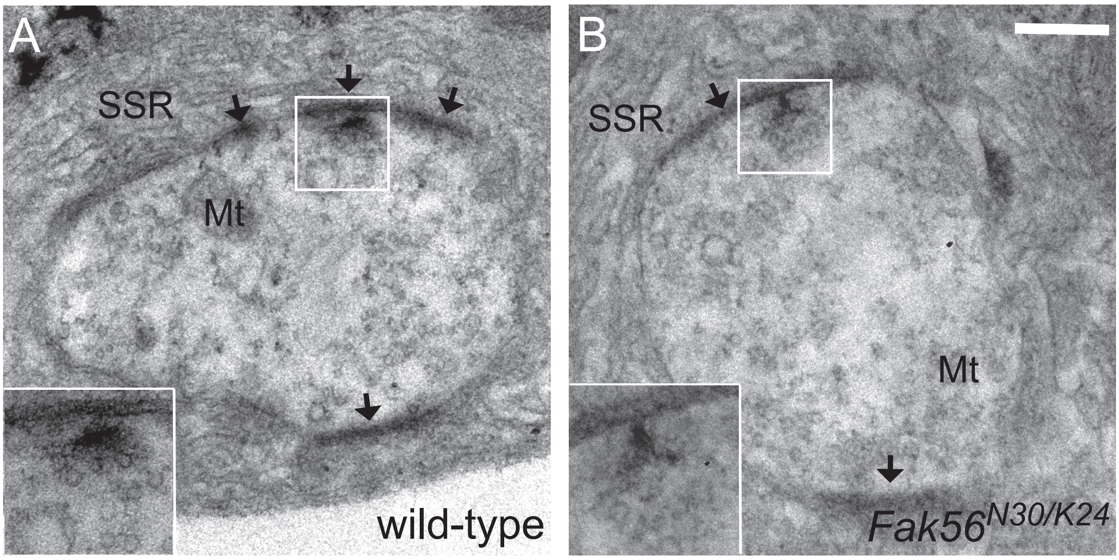

C

	wild-type (n=30)	*Fak56*$^{N30/K24}$ (n=30)	P-value
**Single bouton area** (μm^2)	2.61 ± 0.55	2.60 ± 0.27	0.940
**Bouton perimeter** (μm)	6.02 ± 0.53	6.04 ± 0.35	0.976
**Active zone / Bouton**	4.15 ± 0.50	3.6 ± 0.29	0.349
**Total active zone length** (μm) **/ Bouton**	1.95 ± 0.31	2.05 ± 0.18	0.776
**Total active zone length** (μm) **/Bouton perimeter** (μm)	0.30 ± 0.03	0.34 ± 0.02	0.279
**T bar number / Bouton**	1.45 ± 0.31	1.27 ± 0.20	0.623
**Bouton area** (μm^2) **/Active zone**	0.60 ± 0.10	0.79 ± 0.08	0.150
**T bar number /Active zone**	0.32 ± 0.07	0.37 ± 0.05	0.642
**Vesicle number /Bouton area** (μm^2)	81.86 ± 9.06	84.46 ± 7.62	0.827

Additional file 3. (A-B) Electron micrographs of cross-sections through a type I bouton of muscle 6/7 in wild-type and *Fak56*$^{N30/K24}$ larvae. Squares show active zones with a T bar, which are enlarged in lower-left corner. Subsynaptic reticula (SSR), active zones (arrow), and mitochondria (Mt) are indicated. Scale bar, 2μm. (C) Features of the synaptic ultrastructure were quantified, and no significant difference in these parameters was found between wild-type and *Fak56*$^{N30/K24}$. Dissected larval body walls (including the CNS and motor axons) were fixed at RT for 30 minutes, followed by 4°C overnight in modified Trump's fixative (0.1 M sodium cacodylate buffer, 1% glutaraldehyde, and 4% formaldehyde). The fixed specimens were rinsed three times in 0.1 M sodium cacodylate buffer for 10 minutes, post-fixed for 30 minutes with 2% osmium tetroxide in

0.1 M sodium cacodylate buffer, rinsed three times for 10 minutes in 0.1 M sodium cacodylate buffer, and finally rinsed five times in ddH_2O for 10 minutes. The muscle 6/7 in the A3 segment was knife-dissected out, and specimens were then stained *en bloc* in 2% aqueous uranyl acetate for 20 minutes, dehydrated in a graded ethanol series, and subsequently set into the Spurr's embedding medium. Thin sections (90 nm) were stained with uranyl acetate and lead citrate, and images were viewed on a Tecnai G2 Spirit TWIN electron microscope (FEI Company) and captured on a Gatan CCD Camera (794.10.BP2 MultiScan™). TEM data were quantified by MetaMorph V6.3r7 (Molecular Devices).
