# Supplementary material for: Fak56 functions downstream of integrin alphaPS3betanu and suppresses MAPK activation in neuromuscular junction growth
Source: Neural Dev. 2008 Oct 16;3:26. doi: 10.1186/1749-8104-3-26 (PMC2576229; doi:10.1186/1749-8104-3-26)
Supplement: Additional file 4 — Electrophysiological recording of postsynaptic currents from wild-type and Fak56N30/K24 in 1 mM [Ca2+]. (A) Cumulative frequency plot reveals a significant shift in the distribution of mEJP amplitudes. (B) Representative traces and mean amplitudes of EJPs in wild-type and Fak56N30/K24. [file 1749-8104-3-26-S4.pdf]

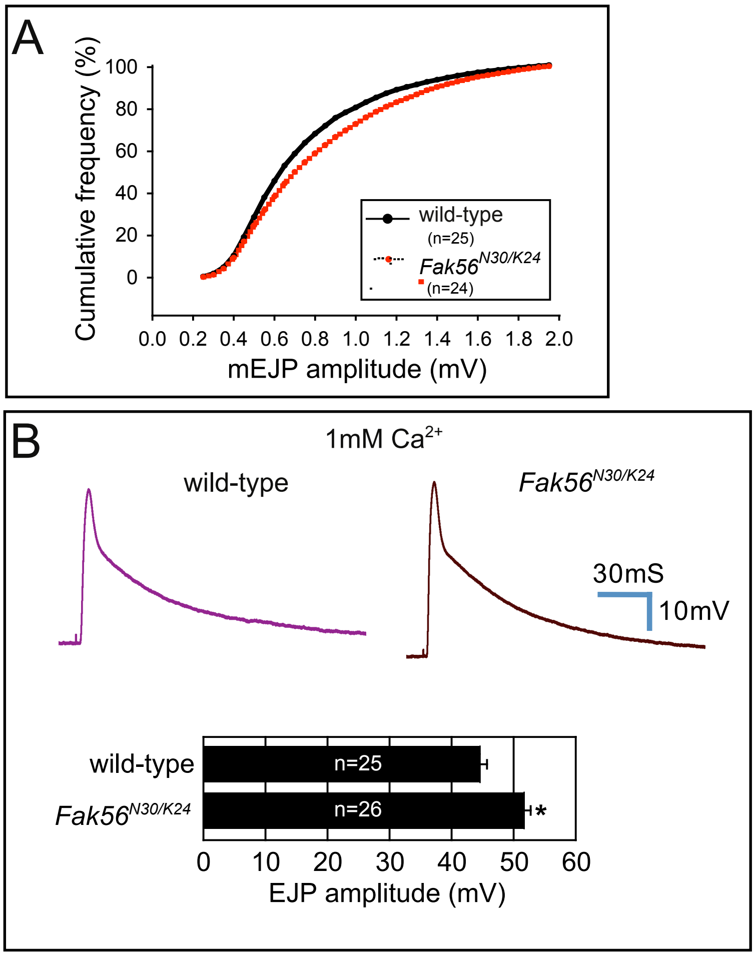

Additional file 4. Electrophysiological recording of postsynaptic currents from wild-type and *Fak56*$^{N30/K24}$ in 1 mM [Ca^{2+}]. Muscle 6 of segment A3 in late 3rd instar larvae was recorded. (A) Cumulative frequency plot reveals a significant shift in the distribution of mEJP amplitudes. Note that the skews of cumulative frequency plot decreased in *Fak56*$^{N30/K24}$ (1.6 ± 0.1) as compared to wild-type (2.3 ± 0.3, $0.01<p<0.025$ by Kruskal-Wallis *h* test). There was no statistically significant changes between wild-type and *Fak56* mutant in the frequency of mEJPs (0.98±0.11 vs. 1.20±0.08, p=0.1159 by Student *t* test), in the variance/mean of mEJPs (0.28±0.06 vs. 0.31±0.03, $0.01<p<0.025$ by Kruskal-Wallis *h* test) and in the membrane resting potential (-72.2±1.4 mV vs. -75.4±1.0 mV, p=0.076 by Student *t* test). (B) Representative traces and mean amplitude of EJPs in wild-type and *Fak56*$^{N30/K24}$. Note that a significant increase of the mean EJP amplitude was detected in *Fak56*$^{N30/K24}$ as compared to wild-type ($p<0.0001$ by Student *t* test). Calibration: 30 ms, 10 mV for evoked release.
