# Supplementary material for: Fak56 functions downstream of integrin alphaPS3betanu and suppresses MAPK activation in neuromuscular junction growth
Source: Neural Dev. 2008 Oct 16;3:26. doi: 10.1186/1749-8104-3-26 (PMC2576229; doi:10.1186/1749-8104-3-26)
Supplement: Additional file 5 — BMP/Gbb signaling-independent mechanism of Fak56 in NMJ growth. No alternations of NMJ phenotypes were detected by introducing mutant alleles (sax4, witA12 and med13) for BMP signaling components into elav>Fak56RNAi. [file 1749-8104-3-26-S5.pdf]

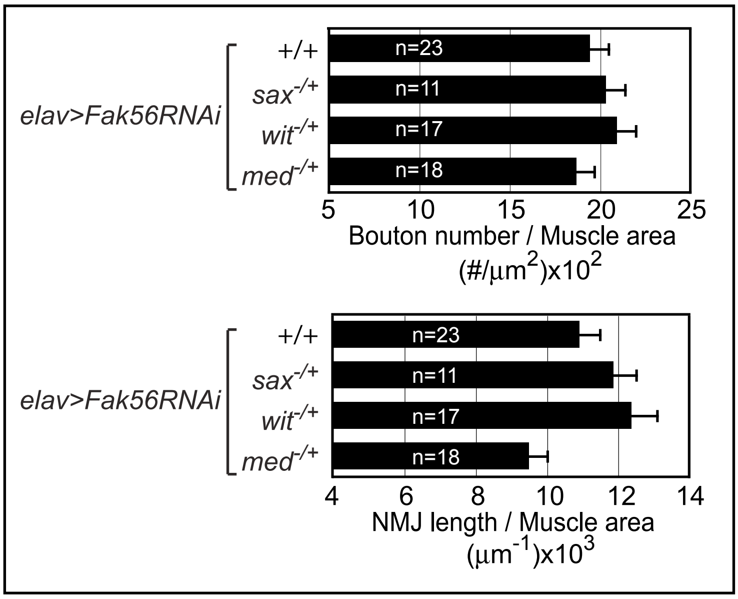

Additional file 5. BMP/Gbb signaling-independent mechanism of Fak56 in NMJ growth. Quantification of NMJ 6/7 phenotypes for the control *elav>Fak56RNAi* (same set of data from Figure 5), *elav>Fak56RNAi;sax*$^{4/+}$, *elav>Fak56RNAi;wit*$^{A12/+}$ and *elav>Fak56RNAi;med*$^{13/+}$. Note that no significant phenotypic alternations observed at NMJs by introducing one null allele of BMP signaling components into *elav>Fak56RNAi.* Statistics was done as in Figure 1E.
