# Supplementary material for: Fak56 functions downstream of integrin alphaPS3betanu and suppresses MAPK activation in neuromuscular junction growth
Source: Neural Dev. 2008 Oct 16;3:26. doi: 10.1186/1749-8104-3-26 (PMC2576229; doi:10.1186/1749-8104-3-26)
Supplement: Additional file 6 — ERK phosphorylation in Fak56CG1 mutant embryos. Expressions of phospho-ERK appear grossly normal during Drosophila embryogenesis. [file 1749-8104-3-26-S6.pdf]

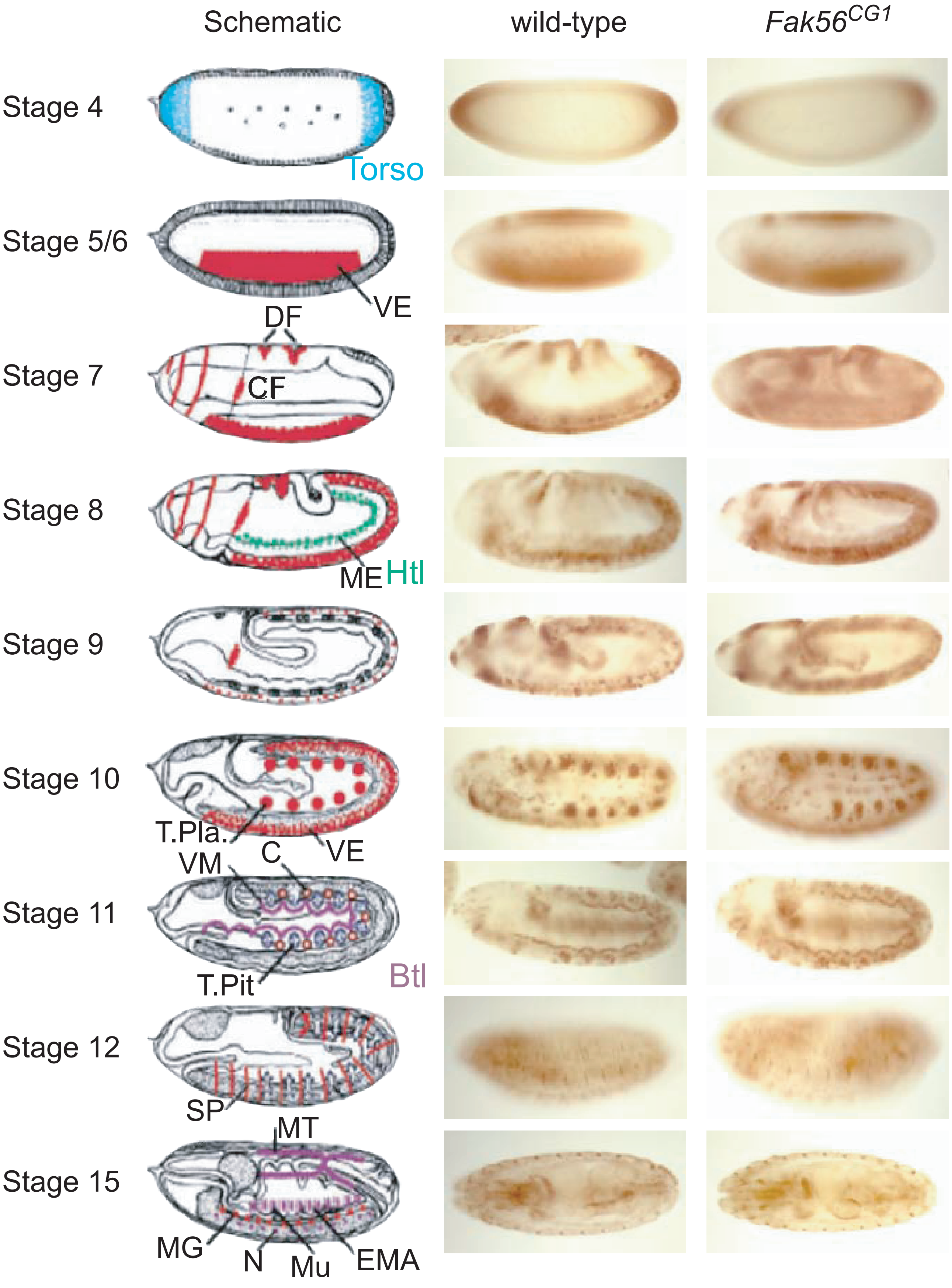

Schematic
wild-type
Fak56CG1
Stage 4
Torso
Stage 5/6
VE
DF
Stage 7
CF
Stage 8
ME
Htl
Stage 9
Stage 10
T.Pla.
VE
VM
C
Stage 11
T.Pit
Btl
Stage 12
SP
MT
Stage 15
MG
N
Mu
EMA

Additional file 6. ERK phosphorylation appears grossly normal during *Drosophila* embryogenesis. *Fak56*^{CG1} and wild-type embryos were analyzed with anti-dpERK antibodies and compared with the *Drosophila* atlas of MAPK activation patterns during embryogenesis [1], to analyze the requirement for *Fak56* downstream of identified RTKs.

References:

1. Gabay L, Seger R, Shilo BZ: **In situ activation pattern of Drosophila EGF receptor pathway during development.** *Science* 1997, **277:**1103-1106.
